# Influence of War on Births

**Published:** 1915-10

**Authors:** 


**Influence of War on Births.** During the quarter year ending July 1, the births in Berlin were 7,523, as compared with 10,030 for 1915. For England and Wales, the births numbered 213,094, 12,973 below last year's number for the same quarter. Deaths in England and Wales were 138,579, 14,445 more than in the same quarter of 1914.

